# The importance of appraising articles and conducting psychiatric journal clubs

**DOI:** 10.1177/10398562231191678

**Published:** 2023-07-25

**Authors:** Edward Miller, Michael James Weightman, Ashna Basu, Andrew Amos

**Affiliations:** School of Medicine, 340864 The University of Auckland, Auckland, New Zealand; School of Medicine, 1066 The University of Adelaide, Adelaide, SA, Australia;; and College of Medicine and Public Health, Flinders University, Adelaide, SA, Australia; 6804 Prince of Wales Hospital, Sydney, NSW Australia;; Discipline of Psychiatry and Mental Health, UNSW, Sydney, NSW, Australia;; and Committee for Research, Royal Australian and New Zealand College of Psychiatry, Melbourne VIC, Australia; School of Medicine and Dentistry, 104397 James Cook University, Townsville, QLD, Australia

**Keywords:** psychiatric education, journal club, basic science

## Abstract

**Objective:**

To describe the importance of scientifically rigorous journal clubs in psychiatric education, and to provide a framework to effectively run journal clubs and appraise articles in a journal club format. This paper explores the concept of journal clubs and describes issues with the current state of academic science. It then lists factors associated with effectiveness of journal clubs and outlines a structure for appraising articles relevant to psychiatric practice in a journal club format.

**Conclusions:**

Current models of academic research and publishing, which can reward practices vulnerable to multiple forms of bias, make the consistent and valued use of journal clubs in psychiatric education and continuing professional development more important than ever. The literature shows that journal clubs can provide a valuable forum for mental health clinicians to update themselves on recent medical and scientific knowledge, while practicing and teaching skills in critical appraisal of research, statistics, clinical decision-making and epidemiology.

The current state of academic science means it is more important than ever for researchers and clinicians to value expertise in critically appraising scientific literature. Evidence-based medicine integrates understanding of the scientific method with individual experience as core skills in developing and maintaining high-quality clinical practice, excellent clinical standards and professionalism. This paper briefly describes the background of journal clubs before exploring the current state of academic science, highlighting that critically appraising research remains a vital skill. It will then list factors associated with increased effectiveness of journal clubs as well as describing a structure for how to appraise articles in a psychiatric journal club format.

## Characteristics and benefits of journal clubs

The first documented journal club began in 1835 with a group of students reading journal articles in a room above a baker’s shop in London.^1^ Contemporary journal clubs have typically lasted an hour a week, in a clinical or professional setting and have included not just psychiatrists but also others members of the multi-disciplinary team (MDT), of any seniority, from medical students to consultants or even professors.^2^ Papers for discussion are usually sourced from contemporary or historical peer-reviewed journals, books or case studies. Material is often shared before the discussion so that others can familiarise themselves with the content, and journal club proceedings have been published in scientific journals.^2^ For mental health clinicians, journal clubs may also provide opportunities to discuss psychotherapy cases or key theoretical constructs, and wider psycho-social, philosophical political or service-level aspects which may be important to understanding the complexities of psychiatric issues. Discussions around other factors relevant to psychiatric practice, such as ethics, gender, lived experience and stigma, can also occur.^3^

When done well, journal clubs can be highly intellectually and professionally stimulating for the participants involved. However, there is evidence that psychiatric journal clubs can differ in their rigour, consistency and attendance. One survey of 141 general psychiatric residency programs in the United States found that only 27% offered effective journal clubs, with 14% offering no journal club.^4^ Only 28% held these meetings weekly, and only 38% described the content as being ‘broad based’ (covering all aspect of a bio-psycho-social model). Another small study of 12 junior psychiatric trainees found that participation in a psychiatric residency journal club over a period of 12 months resulted in an increased rate of both perusing and reading psychiatric scientific journals, and also residents being satisfied at their level of comprehension.^5^

## Current challenges within academic science

The significant and escalating concerns regarding the apparent crisis state of modern academic science are explored below and help illustrate the crucial importance of journal clubs.^6^

### Issues with the academic process

It has been argued that current models of academic advancement, scientific research and publishing encourage widespread ‘questionable research practices', which include selective reporting, outcome switching, reciprocal gift authorships, ‘p-hacking’, and – most egregiously – outright fabrication of data.^6,7^ Evidence of declining research integrity has been presented as a serious threat to the efficiency and effectiveness of science for accurately understanding human health and behaviour. The rapid expansion of impact metrics such as the researcher h-index or journal impact factor can also introduce additional biases. For instance, the h-index (a measure of an individual author’s impact by calculating the ratio of published articles to citations) includes self-citations, which can result in a higher score when an author repeatedly cites their own work.^8^ Recently the entire editorial board of *Neuroimage*, an imaging neuroscience journal, resigned over ethical issues regarding the publication fee and profit margins set by the parent publisher, Elsevier, suggesting that the journal may be using the high impact factor to justify the large fees involved.^9^

### Methodological issues in research

Many research findings are thought to be incorrect, mainly due to low pre-test probability and bias included in research design, data, analysis and presentation.^10^ This is in addition to the use of small sample sizes, underpowered studies, prospective changeability in study design, financial and prejudicial bias (such as repeatedly conducting research in high-income countries) and the popularity of a given subject area.^10^ Bias is also introduced in the publishing process, as studies with significant or ‘positive’ results are more likely to be published than studies with negative or inconclusive results.^11^ Publication bias may also, in part, contribute to the current ‘replication crisis’ in science, whereby many ostensibly ‘positive’ findings are difficult to reproduce.^10^ The focus on statistical significance is particularly problematic in the medical field, as statistical significance is usually less important to clinical practice than clinical significance (such as through measurement of effect size).^12^ However, effect size is also subject to publishing bias, as journals are still more likely to publish results with large effect sizes.^13^

### Issues with research volume

The exponentially increasing rate of production of medical research has also created a significant information overload – for example, there were at least 142 new clinically based Randomised Controlled Trials (RCTs) published every day in the first 5 months of 2020.^14^ Finding relevant articles, letting alone taking the time to appraise them, can be impossibly time consuming.

Intense competition for grant funding and academic posts can contribute to the well-established ‘publish or perish’ culture, whereby academics may feel pressured to publish as much material as possible, regardless of its inherent quality, value or integrity, to boost their profile and increase their chances of promotion and further grant funding.^15^ These problems are all at least in part due to pressures within the academic community. For example, only 0.5% of people in the UK with PhDs ever become professors, and in the USA, only 12.8% achieve academic tenure.^6^ Journals may also compete for publications that will be highly cited, for example, on a topical subject area such as COVID-19 research.^6^ However, since the pandemic began, there have been at least 303 article retractions in mainstream medical and science journals, with many more having documented expressions of concern.^16^ Organisations such as ‘Retraction Watch’ are one way to keep track of the data on article retractions and the reasons given.^17^

### Enhancing research integrity and promoting scientific literacy

Professional bodies such as the Royal College of Psychiatrists have responded to current problems in academic medicine with initiatives like the Research Integrity Group, which advises the Editorial Boards of the *BJPsych*, *BJPsych Open* and *BJPsych Bulletin*.^6^ The group provides improved oversight following allegations of poor research integrity in the journals’ published output. These journals have also tightened checks on ethics permission, trial methodology and plagiarism screening. Psychiatric journal clubs can function as a related tool for students, junior and senior clinicians to practice and refine their critical appraisal and research skills in the context of discussing new or emerging evidence or scientific results.

## Elements of a successful journal club

Multiple factors for maximising the effectiveness of a journal club were detailed in a comprehensive earlier review by Deenadayalan et al., adapted here as Table 1.^18^ Key considerations include ensuring the articles selected meet the specific needs of the group members, establishing effective leadership at each meeting and maintaining a clear routine around the chosen format of the journal club. A similar study by Yager et al. reported that the most effective psychiatric journal clubs: (i) were held frequently in a convenient location with required expectations to attend; (ii) encouraged the presence of the department chair and training director; (iii) discussed original research articles with an emphasis on research methods; and (iv) provided instructions on how to read and interpret the literature.^4^

**Table 1.** Factors associated with increased effectiveness of journal clubs

Purpose	• Clear goals/objectives for the journal club that are agreed to by all participants
	• Review goals/objectives regularly and always link to chosen paper at each meeting
Membership	• A critical mass of members is required to establish viability and optimise discussion
	• Appoint a co-ordinator for the group responsible for administrative tasks (e.g. booking venue, correspondence with members)
	• Ensure there are members with sufficient expertise in statistics or research
Format	• Meet at regular time intervals (e.g. monthly)
	• Select mutually appropriate time to maximise attendance
	• Encourage regular attendance and ensure an attendance record is maintained
	• Each meeting requires a leader to present the article and lead discussion (the individual in this role can change from meeting to meeting)
Article selection	• Ensure input of members for article selection, either through consensus system or use of rotating roster
	• Selected articles must be relevant to the group’s stated purpose
	• Circulate chosen article sufficiently in advance to allow members to come prepared (e.g. one week in advance)
Clinical relevance	• Ensure discussion of chosen article addresses relevance for clinical practice

Table 2 presents a concise overview of a structure that one might use when presenting and appraising an article in a psychiatric journal club format.

**Table 2.** Proposed structure for presenting and appraising literature in a psychiatric journal club format

Rationale for choosing the article	Why you chose this paper
	• Is there a recent clinical scenario or case which lead you to go to the literature for more information? If so, you could do a short case presentation to provide more of the clinical context
	• Is it helping you to refresh or learn about a particular area prior to an exam, such as statistics?
	• Or an article may be a particular intellectual interest or passion of yours
	How you identified the article
	• Document the search strategy used, or whether the article was already known to you from prior reading
	• Was it recommended by someone, or referenced in another article you were reading?
Article information	• Title of article
	• Name of the journal
	• Publication date
	• The authors – who are they, where do they work, what is their background? (Have a brief look/internet search – what is their other work?)
	• Funding statement – it one present? Who is funding the work?
	• Conflict of interest – have the authors declared any? If so, what conflicts may this present?
	• You could also comment on article metrics, for example, through altmetrics (https://www.altmetric.com)
Background and context	• Have the authors introduced the general background and context to the (upcoming) research questions/aims?
	• Have they included a brief literature review (or the article itself may be a literature review)?
	• Is the context clear – have they justified the basis and value of the research question?
	∘ This could be done in the PICO format – patient, population or problem, intervention, comparison and outcome
Aims	• Are the aim(s) clearly stated?
	• Do they match the background and context?
	• Are they of clinical value (as defined by the background)?
Methodology	• What is the methodology used – is it justified by the authors?
	• If the article is using a **qualitative** approach, does it address the specific theoretical perspective used – for example, interpretative phenomenological analysis (IPA) or grounded theory?
	∘ Are there appropriate quality checks involved – for example, a bracketing interview, and comments on the coding process, number of coders involved and how disagreements were mediated?
	• If the article is using a **quantitative** approach, has it described the type of data examined (e.g. parametric or non-parametric) and justified the statistical test chosen?
	∘ Has it included various quality and bias checks – such as describing the randomisation process, and was the study blinded?
Results	• What were the key results?
	• Comment on various features and presentation of the results
	• Qualitative
	∘ Are themes presented clearly?
	∘ Are key quotes included?
	• Quantitative
	∘ Are results statistically significant?
	∘ Are *p* values and effect sizes provided?
	∘ Are the results presented in a clear way, for example, forest plot for a meta-analysis?
Discussion	• Do the authors provide a discussion, interpretation or explanation of the results?
	• Do they link it in with prior or existing literature?
	• Do they explain or justify any anomalous or unexpected results?
	• Do they address limitations with the study?
Conclusions	• Are the conclusions valid and in keeping with the evidence provided within/by the study?
	• Are any future questions or directions for research, as a result of the study, provided?
	• Further readings
	∘ Have other authors/experts provided responses to the article that could help interpret the results – for example, through the science media centre (https://www.sciencemediacentre.org/)?

To conclude, current models of academic research and publishing, which can reward practices vulnerable to multiple forms of bias, make the consistent and valued use of journal clubs in psychiatric education and continuing professional development more important than ever. This paper has highlighted reasons for why this is so, alongside suggesting frameworks for how to run journal clubs and critically appraise articles in a journal club format.
